# Supplementary material for: Generation of a humanized Aβ expressing mouse demonstrating aspects of Alzheimer’s disease-like pathology
Source: Nat Commun. 2021 Apr 23;12:2421. doi: 10.1038/s41467-021-22624-z (PMC8065162; doi:10.1038/s41467-021-22624-z)
Supplement: Supplementary file 1 — Supplementary Information [file 41467_2021_22624_MOESM1_ESM.pdf]

Supplementary Information

Generation of a humanized Aβ expressing mouse demonstrating aspects of Alzheimer's disease-like pathology

Baglietto-Vargas et al.

a

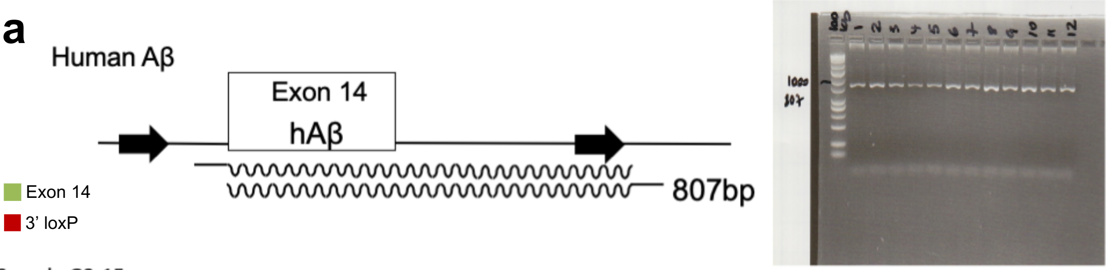

Sample G2-15:

NNNNNNNANNNGNNCTGCNTACTTTGTGNTTGACGCAGGTTCTGGGCTGACAAACATCAAGACGGAAGAGATCTCGGAAGTGAAGATG
GATGCAGAATTCAGACATGATTCAGGATATGAAGTCCACCATCAAAAACTGGTAGGCAAAAATAAACTGCCTCTCCCCGAGATT
GCGTCTGGCCAGATGAAATACGTGGCACCTCGTGGCTTGTCCTGTGTCAAAACTGAGAAGGACTACTGGGAATAAAACCAAAATGCCTGCCT
AGATCTTCACAAAGATAGGAAGGAGAGGAAGTGGGGCTCTGTTGATAGTTCTTGCTGAGCAGAAGCCGCTGAGCCCAGGCGGAACATACA
AGTGTAATTCAGTCCAACGTTGCAGCTACGTGAGCTGACTTCCTAGGAAAATGGTTTTTTCGGGTTAAACACATCTAATCCCAATATCCAAACT
GGAAGGCAGGATACCACAAACAAGAAATTCAAGTCCATGAGGCTCTGATATTACTGTGTGGGGGAAGCATAAGTGAATTTCATTTAGAGGTG
GTCCCAGTTTCCAAGATGGTCTCATGAACTGATGGCGAGCTCAGACCATAACTTCGTATAATGTATGCTATACGAAGTTATCATTAATT
GCGTTGCGCCATCTCTATGTAAATACCAAAAAAAAAAAAAAAGTTAGAAATCAAGATTTTCGAAACCCTTTTGGTCATGACCATTCTCGGTCCAC
GATAGTTTTATTTGAANAATTGNNCTTAANTAANCATGNGNNGGNNNNNNNNNNNNCTNGNN

Sample H3-24:

NNNNNANNNNNGNNNNGCNTACTTTGTNNTTGACGCAGGTTCTGGGCTGACAAACATCAAGACGGAAGAGATCTCGGAAGTGAAGATG
GATGCAGAATTCAGACATGATTCAGGATATGAAGTCCACCATCAAAAACTGGTAGGCAAAAATAAACTGCCTCTCCCCGAGATT
GCGTCTGGCCAGATGAAATACGTGGCACCTCGTGGCTTGTCCTGTGTCAAAACTGAGAAGGACTACTGGGAATAAAACCAAAATGCCTGCCT
AGATCTTCACAAAGATAGGAAGGAGAGGAAGTGGGGCTCTGTTGATAGTTCTTGCTGAGCAGAAGCCGCTGAGCCCAGGCGGAACATACA
AGTGTAATTCAGTCCAACGTTGCAGCTACGTGAGCTGACTTCCTAGGAAAATGGTTTTTTCGGGTTAAACACATCTAATCCCAATATCCAAACT
GGAAGGCAGGATACCACAAACAAGAAATTCAAGTCCATGAGGCTCTGATATTACTGTGTGGGGGAAGCATAAGTGAATTTCATTTAGAGGTG
GNCCCAGTTTCCNAGATGGTCTCATGAACTGATGGCGAGCTCAGACCATAACTTCGTATAATGTATGCTATACGAAGTTATCATTAATT
GCGTTGCGCCATCTCTATGTAAATACCANNNANANANAAAAGNNANAAATCAANATTTTCNAAACCCTTTTGNNCNTNNCCNTNNTCNNCC
NCNACACTTTAATTTTATTACTTGNNNTTNNCTNACNTTGNCAGGAGGGCNAGGGCTNGAAN

Supplementary Figure 1

Supplementary Figure 1. Humanized Aβ sequence design in hAβ-KI mice. a) Sequence analysis of exon 14 confirming that hAβ-KI mice encode humanized non-mutant Aβ (green = Exon14 sequence and red = 3´loxP sequence).

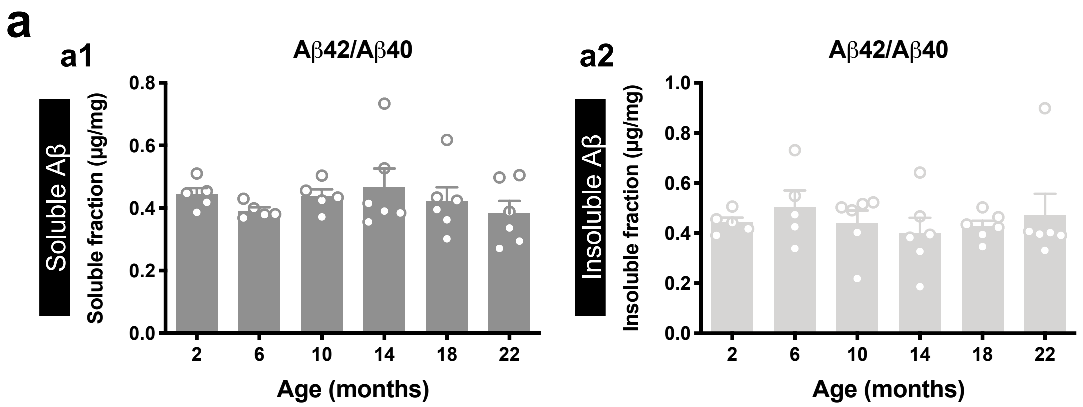

Supplementary Figure 2

Supplementary Figure 2. Soluble and Insoluble 42/40 ratios in hAβ-KI mice. a) Amyloid 42/40 ratios for soluble (a1) and insoluble (a2) Aβ showed similar levels from 2 to 22-month old mice (n=5 in 2mo, 6mo and 10mo and n=6 in 14mo, 18mo and 22mo in the soluble Aβ group. n=5 in 2mo and 6mo and n=6 in 10mo,14mo, 18mo and 22mo in the insoluble Aβ group) (dark-grey = soluble Aβ and light-grey = insoluble Aβ). Data are presented as man values +/- SEM.

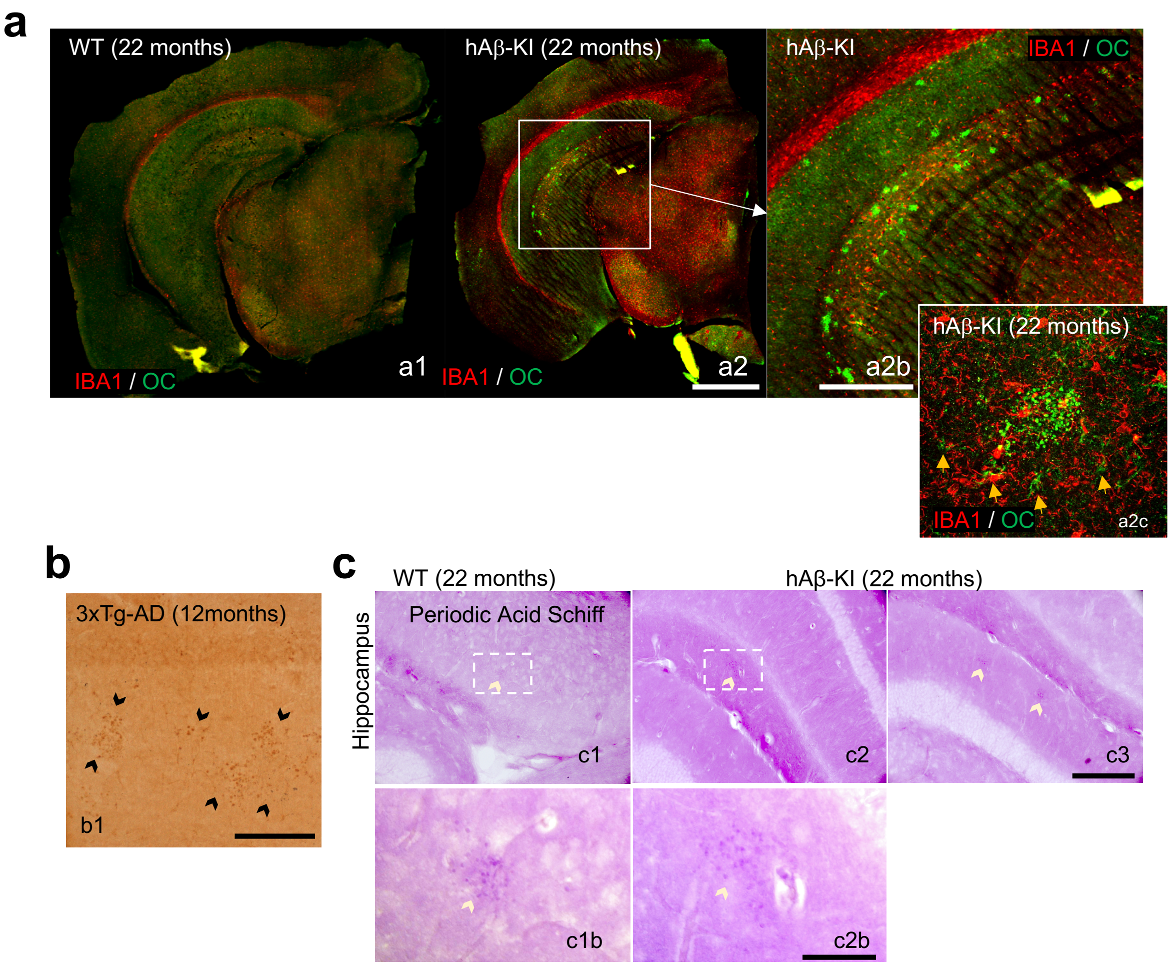

Supplementary Figure 3

Supplementary Figure 3. OC+ clusters of granules in aged hAβ-KI mice. a) Immunostaining for microglia (IBA1, red) and protofibrils (OC, green) in 22-month old WT (a1) and hAβ-KI (a2) mice showing clusters of OC+ clusters associating with the hippocampal fissure (a2, a2b and a2c). A2c) OC+ clusters are composed of numerous small granules, and do not colocalize with microglia, or elicit microglial responses. OC+ cellular processes are seen around the clusters (indicated by yellow arrows). b) Immunostaining for protofibrils (OC) in 12-month old 3xTg-AD mice (indicated by black arrows) c) Periodic Acid Schiff stain in 22-month-old WT (c1 and c1b) and hAβ-KI (c2, c3 and c2b) mouse brains (indicated by yellow arrows).

Scale bar: 1000µm (a1 and a2), 500µm (A2b), 200µm (c1, c2 and c3), 100µm (b1) and 50µm (c1b and c2b).

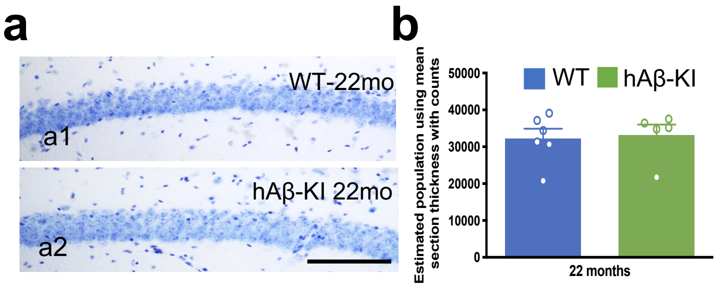

Supplementary Figure 4

Supplementary Figure 4. Neuronal quantification in CA1 hippocampus in hAβ-KI and control mice. a) Representative images of CA1 hippocampal region of the stratum pyramidal in WT (a1) and homozygous hAβ-KI (a2) mice stained with cresyl violet solution. b) Quantification of a using StereoInvestigator software (unpaired, two-tailed t-test, ns p=0.8225) (blue = WT and green = hAβ-KI) (WT n=5 and hAβ-KI n=6) . Data are presented as mean values +/- SEM. Scale bar: 100µm (a1 and a2).

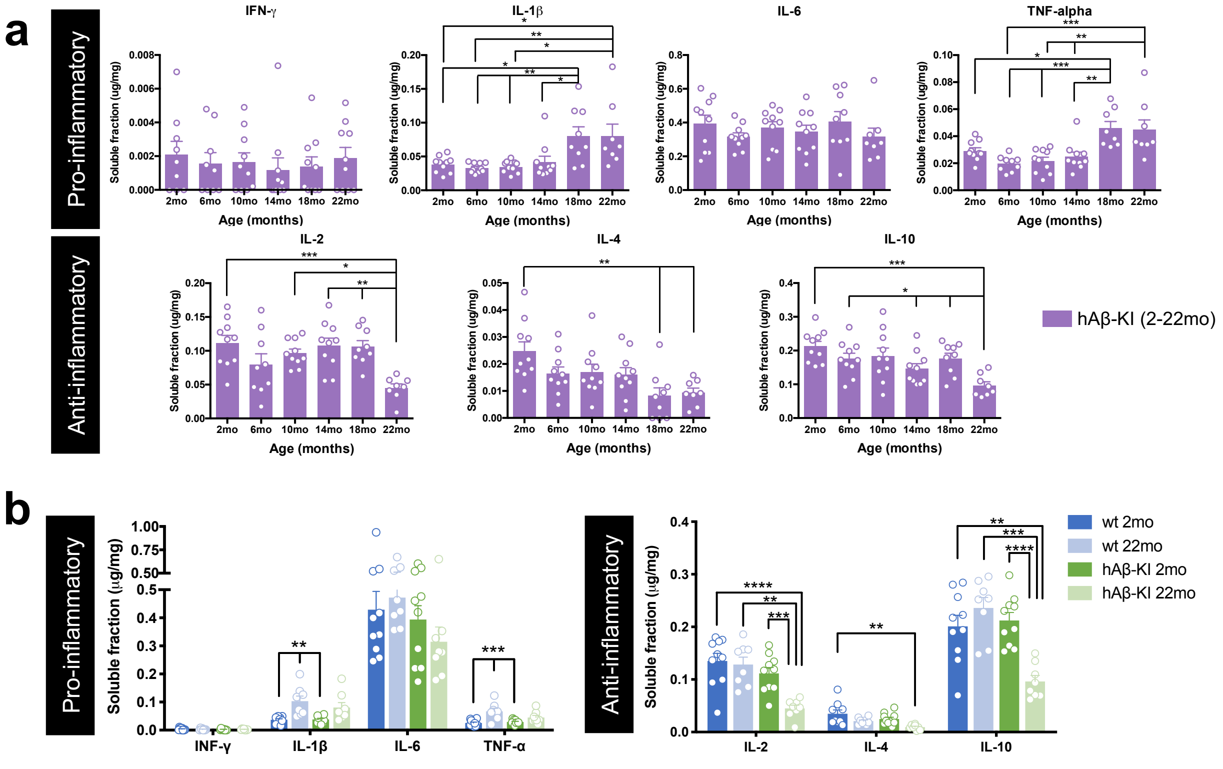

Supplementary Figure 5

Supplementary Figure 5. Inflammatory response in hAβ-KI mice. a) IFN-γ, IL-1β, IL-6, TNF-α, IL-2, IL-4 and IL-10 levels were quantified in hAβ-KI by using the MSD V-PLEX Plus Proinflammatory Panel 1 Mouse Kit (IFN-γ, One-way ANOVA, $F_{5,52}$=0.262, Tukey's post hoc test, ns p=0.931; IL-1β, One-way ANOVA, $F_{5,51}$=5.986, Tukey's post hoc test, *p<0.05 and **p<0.01; IL-6, One-way ANOVA, $F_{5,51}$=0.756, Tukey's post hoc test, ns p=0.585; TNF-α, One-way ANOVA, $F_{5,49}$=8.245, Tukey's post hoc test, *p<0.05, **p<0.01 and ***p<0.001; IL-2, ANOVA, $F_{5,50}$=5.271, Tukey's post hoc test, *p<0.05, **p<0.01 and ***p<0.001; IL-4, One-way ANOVA, $F_{5,51}$=4.422, Tukey's post hoc test, **p<0.01, IL-10, One-way ANOVA, $F_{5,51}$=4.984, Tukey's post hoc test, *p<0.05 and ***p<0.001) (in 2mo group n=10 except IFN-γ n=9, in 6mo group n=10 except IFN-γ, TNF-α, IL-2 n=9, in 10mo group n=10, in 14mo group n=10, in 18mo group n=9, except IFN-γ n=10 and TNF-α n=8, and in 22mo group n=8 except IFN-γ n=10) (purple = from 2mo to 22mo hAβ-KI). b) Cytokine quantification in WT and hAβ-KI mice at 2- and 22-month-old

using the MSD V-PLEX Plus Proinflammatory Panel 1 Mouse Kit (IFN-γ, two-way ANOVA, no main interaction $F_{1,32}$=1.055 or effect in aging $F_{1,32}$=1.055 and genotype $F_{1,32}$=1.055; IL-1β, two-way ANOVA, main effects of age $F_{1,32}$=21.83, Tukey's post hoc test, **p<0.01; IL-6, two-way ANOVA, no main interation $F_{1,32}$=0.756 or effect in aging $F_{1,32}$=0.11 and genotype $F_{1,32}$=3.126; TNF-α, two-way ANOVA, main effects of age $F_{1,32}$=21.71, Tukey's post hoc test, ***p<0.001; IL-2, two-way ANOVA, main effects of genotype $F_{1,32}$=18.78, Tukey's post hoc test, **p<0.01, ***p<0.001 and ****p<0.0001; IL-4, two-way ANOVA, main effects of genotype $F_{1,31}$=5.676, Tukey's post hoc test, **p<0.01; IL-10 two-way ANOVA, main effects of genotype $F_{1,32}$=12.99, Tukey's post hoc test, **p<0.01, ***p<0.001 and ****p<0.0001) (2mo WT group n=10 except IL4 n=9, 22mo WT group n=8, 2mo hAβ-KI group n=10, except IFN-γ n=9 and 22mo hAβ-KI group n=8) (blue = WT 2mo, light-blue = WT 22mo, green = hAβ-KI 2mo and light-green = hAβ-KI 22mo). Data are presented as mean values +/- SEM.

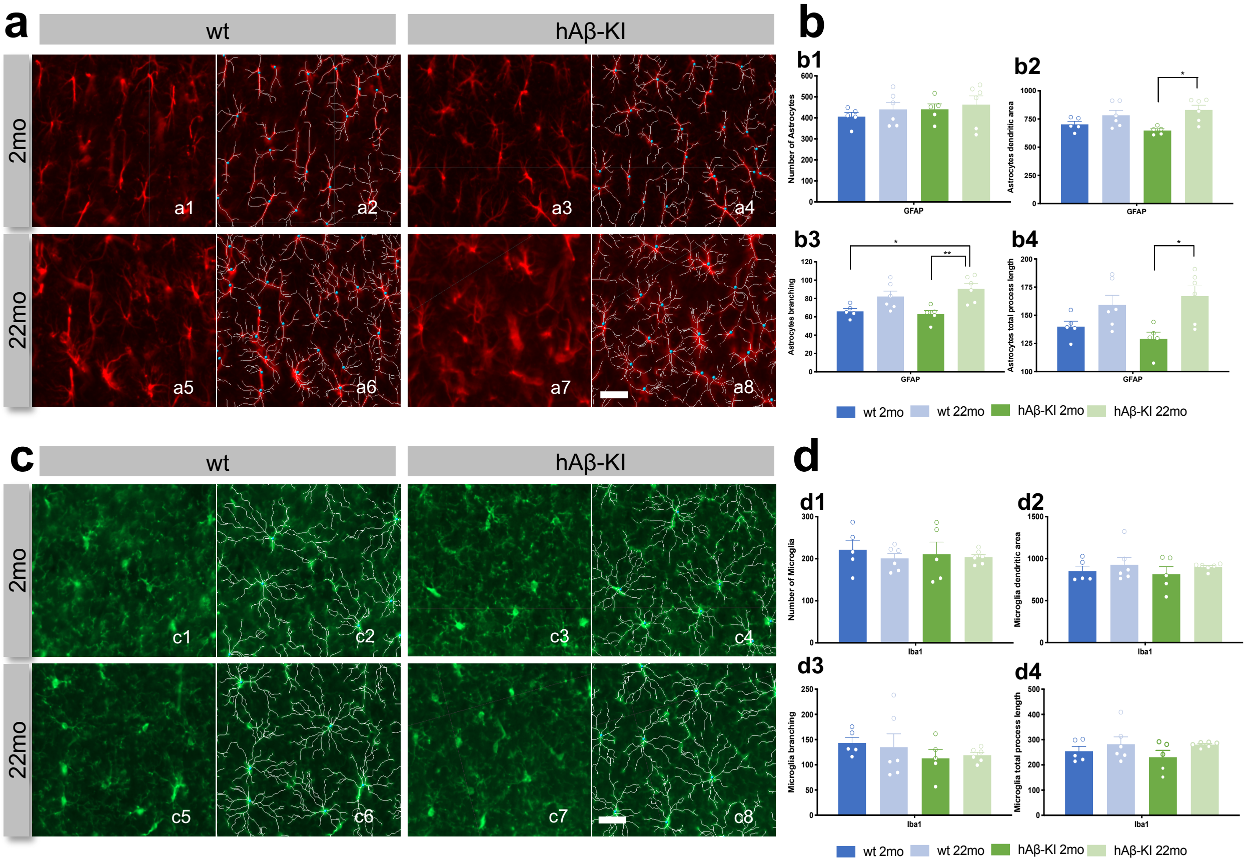

Supplementary Figure 6

Supplementary Figure 6. Microglia and astroglia morphological changes in hAβ-KI mice.

a) Representative images of GFAP staining cells in WT (a1, a2, a5 and a6) and hAβ-KI (a3, a4, a7 and a8) mice at 2- (a1-a4) and 22- (a5-a8) month of age. d) Densitometric analysis using Imaris software to quantify number (b1) (two-way ANOVA, no main interaction $F_{1,19}$=0.38 or effect in aging $F_{1,19}$=1.578 and genotype $F_{1,19}$=1.614), size (b2) (two-way ANOVA, main effects of age $F_{1,18}$=1.986, Tukey's post hoc test, *$p<0.05$), branching (b3) (two-way ANOVA, main effects of age $F_{1,18}$=18.42, Tukey's post hoc test, *$p<0.05$ and **$p<0.01$), and process length (b4) (two-way ANOVA, main effects of age $F_{1,18}$=13.69, Tukey's post hoc test, *$p<0.05$) in 2- and 22-month hAβ-KI and WT mice (n=5 in WT and hAβ-KI at 2mo and n=6 in WT and hAβ-KI at 22mo) (blue = WT 2mo, light-blue = WT 22mo, green = hAβ-KI 2mo and light-green = hAβ-KI 22mo). c) Representative images of Iba1 staining cells in WT (c1, c2, c5 and c6) and hAβ-KI (c3,

c4, c7 and c8) mice at 2- (c1-c4) and 22- (c5-c8) month of age. d) Densitometric analysis using Imaris software to quantify number (d1) (two-way ANOVA, no main interaction $F_{1,19}=0.057$ or effect in aging $F_{1,19}=0.387$ and genotype $F_{1,19}=0.957$), size (d2) (two-way ANOVA, no main interaction $F_{1,14}=1.533$ or effect in aging $F_{1,14}=0.640$ and genotype $F_{1,14}=0.002$), branching (d3) (two-way ANOVA, no main interaction $F_{1,13}=0.277$ or effect in aging $F_{1,13}=1.735$ and genotype $F_{1,13}=0.839$), and process length (d4) (two-way ANOVA, no main interaction $F_{1,17}=0.08$ or effect in aging $F_{1,17}=5.298$ and genotype $F_{1,17}=0.556$) in 2- and 22-month hAβ-KI and WT mice (n=5 in WT and hAβ-KI at 2mo and n=6 in WT and hAβ-KI at 22mo) (blue = WT 2mo, light-blue = WT 22mo, green = hAβ-KI 2mo and light-green = hAβ-KI 22mo). Data are presented as mean values +/- SEM. Scale bar: 30µm (a1-a8 and c1-c8).

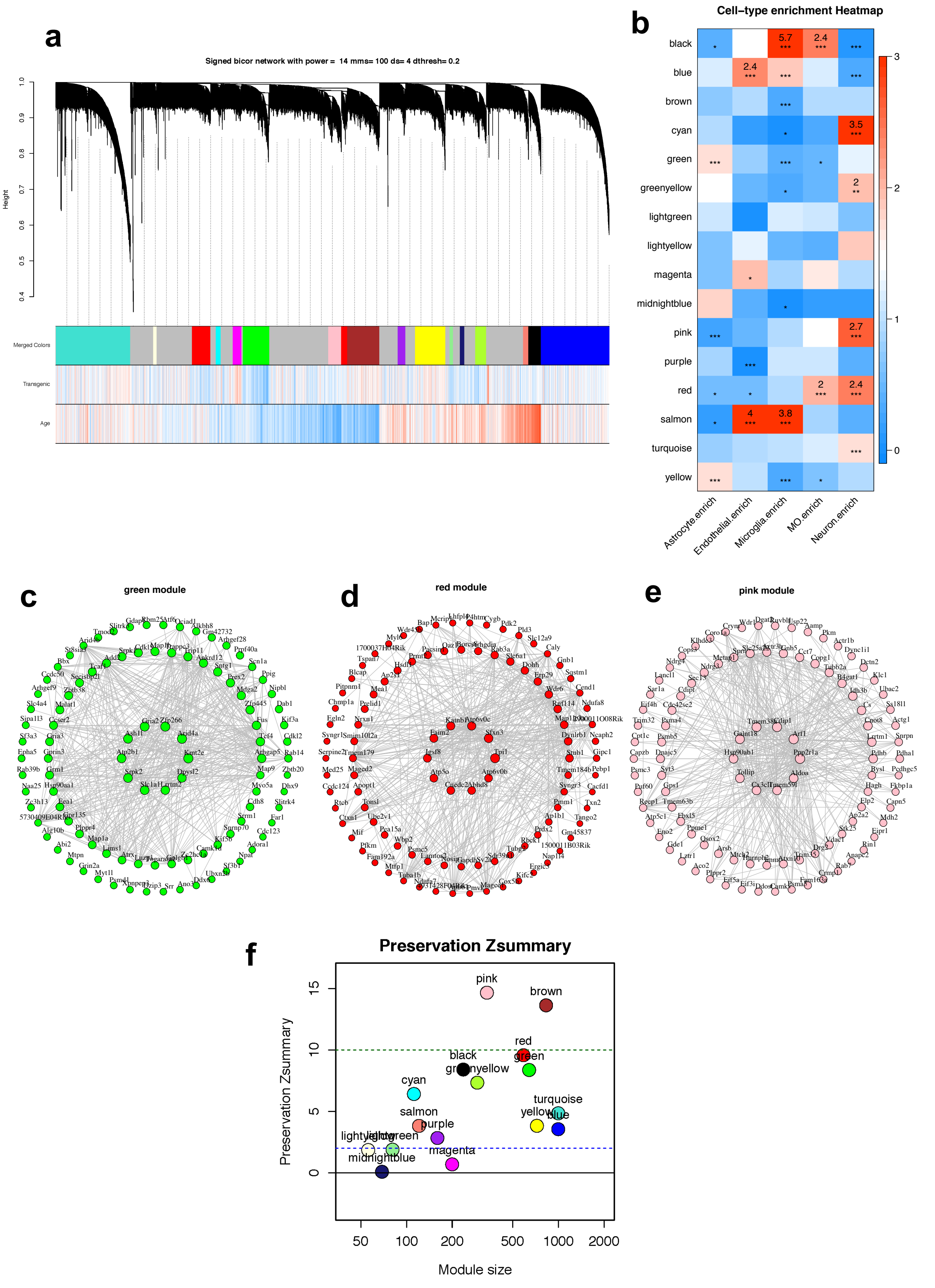

Supplementary Figure 7

Supplementary Figure 7. Co-expression network analysis in hAβ-KI mice. a) WGCNA dendrogram showing modules identified in hAβ-KI mice and the relation of gene-expression with transgenic condition and age. b) Cell-type enrichment analysis showing enrichment of WGCNA modules with known marker genes from each cell-type obtained from Zhang et al., 2014. Values indicate log-fold change of enrichment and FDR corrected q-values are indicated as ***q<0.005; **q<0.01; *q<0.05. c-e) Network plot showing hub genes in center for green (c), red (d) and pink (e) modules. f) Preservation plot showing Zsummary scores obtained from module preservation analysis in WGCNA using Mayo temporal cortex AD data as test data and hAβ-KI mice WGCNA as reference data.

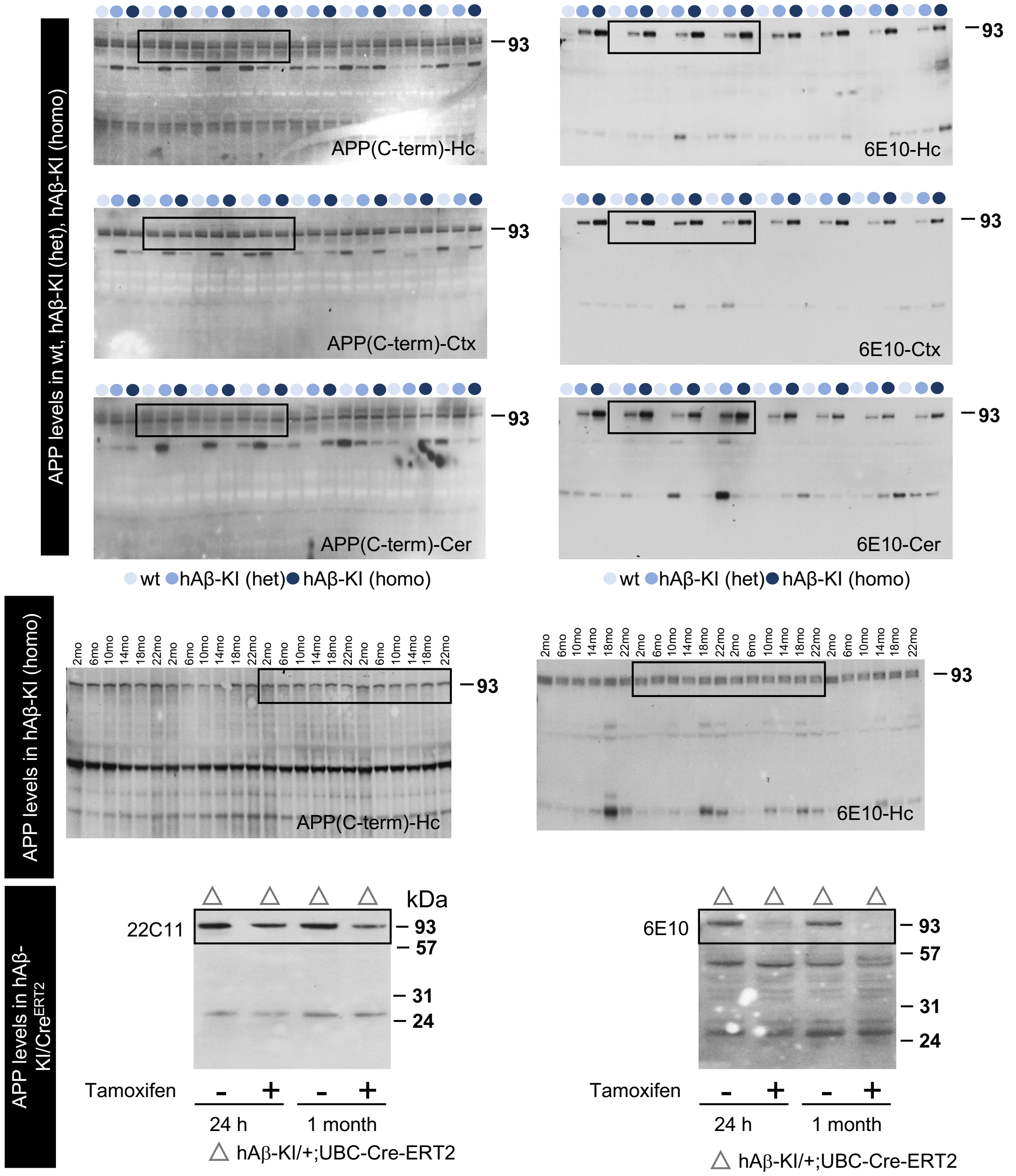

Supplementary Figure 8

Supplementary Figure 8. Full-length western blots. Full-length western blot images used in the study. The rectangular area indicates the area of the blot selected in previous figures. (light-blue = WT, blue = heterozygous hAβ-KI, dark-blue = homozygous hAβ-KI and triangle symbol = hAβ-KI homozygous; UBC-CRE^{ERT2} hemizygous mice).

Supplementary Table 1. Primer pairs for mouse genotyping. List of primer pairs used for animal model genotyping, including hAβ-KI, UBC-Cre-ERT2, PS1-KI and 3xTg-AD mice.

Animal model primers	Sequence (5′⟶3′)
hAβ-KI-F	CTG TGT GGG GGA AGC ATA AG
hAβ-KI-R	AGA GCA TCC CTA CAA CCA AGT
UBC-Cre-ERT2-F	GAC GTC ACC CGT TCT GTT G
UBC-Cre-ERT2-R	AGG CAA ATT TTG GTG TAC GG
PS1-KI-F	CAC ACG CAA CTC TGA CAT GCA CAG GC
PS1-KI-R	AGG CAG GAA GAT CAC GTG TTC AAG TAC
3xTg-AD-APP-F	AGG ACT GAC CAC TCG ACC AG
3xTg-AD-APP-R	CGG GGG TCT AGT TCT GCA T

Supplementary Table 2. Primer pairs for *App* real-time qPCR. List of primer pairs used to measure *App* mRNA in hAβ-KI heterozygous; UBC-Cre^{ERT2} hemizygous mice.

Primers	Sequence (5′⟶3′)
App-F	TCC GTG TGA TCT ACG AGC GCAT
App-R	GCC AAG ACA TCG TCG GAG TAGT
Gapdh-F	AAC TTT GGC ATT GTG GAA GG
Gapdh-R	ACA CAT TGG GGG TAG GAA CA
