# Supplementary material for: Generation of a humanized Aβ expressing mouse demonstrating aspects of Alzheimer’s disease-like pathology
Source: Nat Commun. 2021 Apr 23;12:2421. doi: 10.1038/s41467-021-22624-z (PMC8065162; doi:10.1038/s41467-021-22624-z)
Supplement: Supplementary file 3 — Reporting summary [file 41467_2021_22624_MOESM3_ESM.pdf]

nature research

Corresponding author(s): Drs. Kim Green and Frank LaFerla

Last updated by author(s): Mar 11, 2021

# Reporting Summary

Nature Research wishes to improve the reproducibility of the work that we publish. This form provides structure for consistency and transparency in reporting. For further information on Nature Research policies, see our Editorial Policies and the Editorial Policy Checklist.

## Statistics

For all statistical analyses, confirm that the following items are present in the figure legend, table legend, main text, or Methods section.

| n/a | Confirmed | |
|---|---|---|
| ☐ | ☒ | The exact sample size ($n$) for each experimental group/condition, given as a discrete number and unit of measurement |
| ☐ | ☒ | A statement on whether measurements were taken from distinct samples or whether the same sample was measured repeatedly |
| ☐ | ☒ | The statistical test(s) used AND whether they are one- or two-sided<br>*Only common tests should be described solely by name; describe more complex techniques in the Methods section.* |
| ☒ | ☐ | A description of all covariates tested |
| ☐ | ☒ | A description of any assumptions or corrections, such as tests of normality and adjustment for multiple comparisons |
| ☐ | ☒ | A full description of the statistical parameters including central tendency (e.g. means) or other basic estimates (e.g. regression coefficient) AND variation (e.g. standard deviation) or associated estimates of uncertainty (e.g. confidence intervals) |
| ☐ | ☒ | For null hypothesis testing, the test statistic (e.g. $F$, $t$, $r$) with confidence intervals, effect sizes, degrees of freedom and $P$ value noted<br>*Give P values as exact values whenever suitable.* |
| ☒ | ☐ | For Bayesian analysis, information on the choice of priors and Markov chain Monte Carlo settings |
| ☒ | ☐ | For hierarchical and complex designs, identification of the appropriate level for tests and full reporting of outcomes |
| ☒ | ☐ | Estimates of effect sizes (e.g. Cohen's $d$, Pearson's $r$), indicating how they were calculated |

*Our web collection on statistics for biologists contains articles on many of the points above.*

## Software and code

Policy information about availability of computer code

| | |
|---|---|
| Data collection | RNA-seq: NAC 2.0 Neurodata Acquisition System.<br>Image acquisition: Imaris 9.2.1, StereoInvestigator 64-bit v11, ACT-2U v1.21.41.176, Zeiss slidescanner Axio Z1 (v2.3).<br>Immunoblotting: ImageJ (64-bit).<br>ELISA: Sector Imager plate reader MSD MESO QuickPlex SQ 120<br>RT-qPCR: Bio-Rad CFX Manager 3.1.<br>Cyclic amplification of Aβ misfolding assay: SoftMax Pro 5.4.5 |
| Data analysis | RNA-seq data were analyzed with STAR v.2.5.1b, RSEM v.1.2.22 and edgeR v.3.2.2. Gene ontology (GO) enrichment were analyzed by Metascape 3.0 and PaintOmics 3. Network analysis was performed using weighted gene co-expression analysis (WGCNA) (v1.69) package in R (v3.6.1). Gene-set enrichment analysis was done using enrich R (v2.1) package. RT-qPCR data were analyzed with Bio-Rad CFX manager 3.1. Graphpad Prism 8® software were used for visualization and statistical analysis. |

For manuscripts utilizing custom algorithms or software that are central to the research but not yet described in published literature, software must be made available to editors and reviewers. We strongly encourage code deposition in a community repository (e.g. GitHub). See the Nature Research guidelines for submitting code & software for further information.

## Data

Policy information about availability of data

All manuscripts must include a data availability statement. This statement should provide the following information, where applicable:

- Accession codes, unique identifiers, or web links for publicly available datasets
- A list of figures that have associated raw data
- A description of any restrictions on data availability

The Fastq files and processed data matrices were deposited in GEO with the accession ID GSE116199 (https://www.ncbi.nlm.nih.gov/geo/query/acc.cgi?acc=GSE116199), also the RNA-seq files are available via the AD Knowledge Portal (https://adknowledgeportal.org). The AD Knowledge Portal is a platform for accessing data, analyses, and tools generated by the Accelerating Medicines Partnership (AMP-AD) Target Discovery Program and other National Institute on Aging (NIA)-supported programs to enable open-science practices and accelerate translational learning. The data, analyses and tools are shared early in the research cycle without a publication embargo on secondary use. Data is available for general research use according to the following requirements for data access and data attribution (https://adknowledgeportal.org/DataAccess/Instructions).

For access to content described in this manuscript see: http://doi.org/10.7303/syn24875599.

Full-length western blots are included in Supplementary Figure S7 and source data are provided with this paper. The other datasets generated during the current study are available from the corresponding author upon request since most of the results have been performed at UCI.

# Field-specific reporting

Please select the one below that is the best fit for your research. If you are not sure, read the appropriate sections before making your selection.

[x] Life sciences [ ] Behavioural & social sciences [ ] Ecological, evolutionary & environmental sciences

For a reference copy of the document with all sections, see nature.com/documents/nr-reporting-summary-flat.pdf

# Life sciences study design

All studies must disclose on these points even when the disclosure is negative.

| | |
|---|---|
| Sample size | Sample size was determined according to previous studies from our lab (PMID: 29877034 , PMID: 26077803, PMID: 24176788, PMID: 23312564) |
| Data exclusions | For behavioral analysis: if an animal did not explore both objects during the training phase, then the test was not scored during the test phase and mice were removed. This criteria was used in all group tested. |
| Replication | For most experimental condition, 3-5 sections of the brain were analyzed and at least 5 animals were analyzed per condition. Most of the experiments has been replicated by other members of the lab and all attempts at replication were reproducible. |
| Randomization | All animals were the same gender, age and from the same strain. Animals were randomly assigned to experimental groups; no further randomization in the experimental conditions was employed. |
| Blinding | Behavioral, RNA-seq, LTP and immunohistochemistry studies were blinded. For all other analyses, investigators were not blinded to the conditions of the experiment. |

# Reporting for specific materials, systems and methods

We require information from authors about some types of materials, experimental systems and methods used in many studies. Here, indicate whether each material, system or method listed is relevant to your study. If you are not sure if a list item applies to your research, read the appropriate section before selecting a response.

## Materials & experimental systems

| n/a | Involved in the study | |
|---|---|---|
| [ ] | [x] | Antibodies |
| [x] | [ ] | Eukaryotic cell lines |
| [x] | [ ] | Palaeontology and archaeology |
| [ ] | [x] | Animals and other organisms |
| [x] | [ ] | Human research participants |
| [x] | [ ] | Clinical data |
| [x] | [ ] | Dual use research of concern |

## Methods

| n/a | Involved in the study | |
|---|---|---|
| [x] | [ ] | ChIP-seq |
| [x] | [ ] | Flow cytometry |
| [x] | [ ] | MRI-based neuroimaging |

## Antibodies

| | |
|---|---|
| Antibodies used | Primary antibodies: 6E10 (1:1000; BioLegend, San Diego, CA, USA; Catalog # 83001, Lot #B2261151), anti-APP-CT20 (1:1000; EMD |

Millipore, Burlington, MA, USA Catalog #171610, Lot #D00080225), anti-APP-22C11 (1:1000; EMD Millipore, Burlington, MA, USA, Catalog #MAB348, Lot #2280425), Aβ40 antibody (1:1000; EMD Millipore, Burlington, MA, USA; Catalog #AB5074P, Lot #3062336), Aβ42 antibody (1:1000; EMD Millipore, Burlington, MA, USA; Catalog #AB5078P, Lot #3172448), Iba1 antibody (1:1000 Fujifilm Wako Chemicals, Osaka, Japan; Catalog #019-19741, Lot #PTE0555), GFAP antibody (1:5000 Abcam, Cambridge, MA, USA; Catalog #ab134436, Lot #GR3197612-3), synaptophysin antibody (1:1000 Abcam, Cambridge, MA, USA; Catalog #ab14692, Lot #GR66861-35), amyloid fibrils OC antibody (1:200 Millipore Sigma, Darmstadt Germany; Catalog #AB2286, Lot#3313147), PSD95 antibody (1:1000 EMD Millipore, Burlington, MA, USA; Catalog #1596, Lot #UI287733).

Secondary antibodies: Anti-rabbit IgG (H+L) Secondary antibody, HRP conjugate (1:10000 Invitrogen, Carlsbad, CA, USA Catalog #31460, Lot #RL240411), Anti-mouse IgG (H+L) Secondary antibody, HRP conjugate (1:10000 Invitrogen, Carlsbad, CA, USA Catalog #31430), Biotinilated anti-rabbit; Vector lab, Burlingame, CA, USA, Catalog#BA-1000, Lot#ZG0122), Biotinilated anti-mouse; Vector lab, Burlingame, CA, USA, Catalog#BA-2000, Lot#ZA0409), goat anti-rabbit Alexa-fluor 488 (Invitrogen, Carlsbad, CA, USA; Catalog #A11034, Lot #1937195), goat anti-chicken Alexa-fluor 555 (Invitrogen, Carlsbad, CA, USA; Catalog #A21437, Lot #1964371), goat anti-mouse Alexa-fluor 555 (Invitrogen, Carlsbad, CA, USA; Catalog #A11004, Lot #1218263).

Stains: Congo Red (C6277-25G; Sigma-Aldrich), thioflavin-S (T-1892; Sigma-Aldrich), Amylo-Glo (1:100; Biosensis, Thebarton, South Australia; Catalog #TR-300-AG) and Periodic Acid Schiff (PAS) stain (ACROS Organics, New Jersey, USA; Cat #AC198381000).

Validation

The antibodies used for immunohistochemistry and immunoblot 6E10, Aβ40, Aβ42, OC, APP C-Terminal, 22C11, IBA1, GFAP, SYN, PSD95, GAPDH, as well as the secundary antibodies are widely used and were validated in our previous studies:

Rodriguez-Ortiz CJ., Prieto GA., Martini AC., Forner S., Trujillo-Estrada L., LaFerla FM., Baglietto-Vargas D., Cotman CW & Kitazawa M. (2020). miR-181a negatively modulates synaptic plasticity in hippocampal cultures and its inhibition rescues memory deficits in a mouse model of Alzheimer´s disease. Aging Cell. 2020 Mar;19(3):e13118. doi: 10.1111/acel.13118.

Sanchez-Mejias., Nuñez-Diaz C., Sanchez-Varo R., Gomez-Arboledas A., Garcia-Leon JA, Fernandez Valenzuela JJ., Mejias-Ortega M., Trujillo-Estrada., Baglietto-Vargas D., Moreno-Gonzalez I., Davila JC., Vitorica J & Gutierrez A. (2020). Distinct disease-sensitive GABAergic neurons in the perirhinal cortex of Alzheimer´s mice and patients. Brain Pathol. 2020 Mar;30(2):345-363. doi: 10.1111/bpa.12785.

Forner S., Martini AC., Prieto GA., Dang CT., Rodriguez-Ortiz CJ., Reyes-Ruiz JM., Trujillo-Estrada L., da Cunha C., Andrews EJ., Phan J., Vu Ha J., Chang AVZD., Levites Y., Cruz PE., Ager RR., Medeiros R., Kitazawa M., Glabe CG., Cotman CW., Golde T., Baglietto-Vargas D & LaFerla FM. (2019). Intra- and extracellular A-amyloid overexpression via adeno-associated virus-mediated gene transfer impairs memory and synaptic plasticity in the hippocampus. Sci Rep. 2019 Nov 4;9(1):15936. doi: 10.1038/s41598-019-52324-0.

Martini AC, Gomez-Arboledas A, Forner S, Rodriguez-Ortiz CJ, McQuade A, Danhash E, Phan J, Javonillo D, Ha JV, Tram M, Trujillo-Estrada L, da Cunha C, Ager RR, Davila JC, Kitazawa M, Blurton-Jones M, Gutierrez A, Baglietto-Vargas D, Medeiros R, LaFerla FM. (2019). Amyloid-beta impairs TOM1-mediated IL-1R1 signaling. Proc Natl Acad Sci USA. 2019 Oct 15,116(42):21198-21206. doi: 10.1073/pnas.1914088116.

Trujillo-Estrada L., Nguyen C., Da Cunha C., Cai L., Forner S., Martini AC., Ager RR.., Prieto AG., Cotman CW., Baglietto-Vargas D & LaFerla FM. (2019). Tau underlies synaptic and cognitive deficits for type 1, but not for type 2 diabetes. Aging Cell. 2019 Jun; 18 (3):d12919. doi: 10.1111/acel.12919.

Baglietto-Vargas D., Prieto GA, Limon A, Forner S, Rodriguez-Ortiz CJ, Ikemura k, da Cunha C, Ager RR., Medeiros R, Trujillo-Estrada L, Martini AC, Kitazawa M, Davila JC, Cotman CW, Gutierrez A & LaFerla FM. (2018). AMPA signaling and acting cytoskeleton impairments underlie early synaptic dysfunction in a mouse model of Alzheimer's disease. Aging Cell. 2018 Jun 6:e12791. doi: 10.1111/acel.12791.

Sosna J., Philipp S., Albay III R., Reyes-Ruiz JM., Baglietto-Vargas D., LaFerla FM & Glabe C. (2018). Early long-term administration of the CSF1R inhibitor PLX3397 ablates microglia and reduces accumulation of intraneuronal amyloid, neuritic plaque deposition and pre-fibrillar oligomers in 5xFAD mouse model of Alzheimer's disease. Molecular Neurodegeneration. 2018 Mar 1;13(1):11. doi: 10.1186/s13024-018-0244-x.

Baglietto-Vargas D., Sanchez-Mejias E, Navarro V, Jimenez S, Trujillo-Estrada L, Gomez-Arboledas A, Sanchez-Mico R, Sanchez-Varo R, Vizuete M, Davila JC, Garcia-Verdugo JM, Vitorica J & Gutierrez A. (2017). Dual roles of A in proliferative processes in an amyloidogenic model of Alzheimer's disease. Scientific Report. 2017 Aug 30;7(1):10085. doi: 10.1038/s41598-017-10353-7.

Baglietto-Vargas D., Chen Y, Dongjin S, Ager RR, Rodriguez-Ortiz CJ, Medeiros R, Myczek K, Green KN, Baram TZ & LaFerlaFM. (2015). Short modern-life like stress exacerbates A-pathology and synapse loss in 3xTg-AD mice. J Neurochem. 134(5):915-26. doi: 10.1111/jnc.13195.

DunnHC, Ager RR, Baglietto-Vargas D, Cheng D, Kitazawa M, Cribbs DH and Medeiros R (2014). Restoration of Lipoxin A4 signaling reduces Alzheimer's disease-like pathology in the 3xTg-AD mouse model. J Alzheimer Dis. 43(3): 893-903. doi: 10.3233/JAD-141335.

Rodriguez-Ortiz CJ, Baglietto-Vargas D, Martinez-Coria H, LaFerla FM and Kitazawa M (2014). Up-regulation of miR-181 decreases c-Fos and SIRT-1 in the hippocampus of 3xTg-AD mice. J Alzheimers Disease. 42(4):1229-38. doi: 10.3233/JAD-140204.

Medeiros R., Castello NA., Cheng D., Kitazawa M., Baglietto-Vargas D., Green KN., Esbenshade TA., Bitner RS., Decker MW., and FM LaFerla. (2014). Alpha-7 Nicotinic receptor agonist enhances cognition in aged 3xTg-AD mice with robust plaques and tangles. Am J Pathol. 184(2):520-9. doi: 10.1016/j.ajpath.2013.10.010.

Trujillo-Estrada L., Jimenez S., Vanesa De Castro., Torres M., Baglietto-Vargas D., Moreno-Gonzalez I., Navarro V., Sanchez-Varo R.,

Davila JC., Vizuete M., Gutierrez A and Vitorica J. (2013). In vivo modification of Abeta plaque toxicity as a novel neuroprotective lithium-mediated therapy for Alzheimer's disease pathology. Acta Neuropathol Communications. 12(1):73. doi: 10.1186/2051-5960-1-73.

Baglietto-Vargas D., Kitazawa M., Elaine J Le., Estrada-Hernandez T., Rodríguez-Ortiz CJ., Medeiros R, Green KN., & LaFerlaFM. (2014). Endogenous murine tau promotes neurofibrillary tangles in 3xTg-AD mice without affecting cognition. Neurobiol Dis. 62C, 407-415. doi: 10.1016/j.nbd.2013.10.019.

Medeiros R., Kitazawa M., Passos GF., Baglietto-Vargas D., Cheng D., Cribbs DH., and FM LaFerla. (2013). Aspirin-triggered lipoxin A4 stimulates alternative activation of microglia and reduces Alzheimer disease-like pathology in mice. Am J Pathol. 182(5), 1780-9. doi: 10.1016/j.ajpath.2013.01.051.

Baglietto-Vargas D., Medeiros R, Martinez-Coria H., LaFerlaFM., & Green KN. (2013). Mifepristone alter amyloid precursor protein processing to preclude amyloid beta and also tau pathology. Biol Psychiatry. 74(5), 357-66. doi: 10.1016/j.biopsych.2012.12.003.

Medeiros R, Kitazawa M, Chabrier M, Cheng D, Baglietto-Vargas D, Kling A, Moeller A, Green KN, & LaFerla FM. (2012). Calpain inhibitor A-705253 mitigates Alzheimer's disease-like pathology and cognitive decline in aged 3xTg-AD mice. Am J Pathol. 181(2), 616-25. doi: 10.1016/j.ajpath.2012.04.020.

Sanchez-Varo R., Trujillo-Estrada L., Sanchez-Mejia E., Torres M., Baglietto-Vargas D., Moreno-Gonzalez I., Vanesa De Castro., Jimenez S., Ruano D., Vizuete M., Davila JC., Garcia-Verdugo M., Jiménez AJ., Vitorica J & Gutierrez A (2012). Abnormal accumulation of autophagic vesicles correlates with axonal and synaptic pathology in young Alzheimer's mice hippocampus. Acta Neuropathol. 123 (1), 53-70. doi: 10.1007/s00401-011-0896-x.

Medeiros R, Kitazawa M, Caccamo A, Baglietto-Vargas D, Estrada-Hernandez T, Cribbs DH, Fisher A, & LaFerla FM. (2011). Loss of muscarinic M1 receptor exacerbates Alzheimer's disease-like pathology and cognitive decline. Am J Pathol. 179(2), 980-91. doi: 10.1016/j.ajpath.2011.04.041.

Caccamo A, Oddo S, Billings LM, Green KN, Martinez-Coria H, Fisher A & LaFerla FM. (2006). M1 receptors play a central role in modulating AD-like pathology in transgenic mice. Neuron. 2;49(5):671-82. doi: 10.1016/j.neuron.2006.01.020.

## Animals and other organisms

Policy information about studies involving animals; ARRIVE guidelines recommended for reporting animal research

| | |
|---|---|
| Laboratory animals | B6(SJL)-Apptm1.1Aduci/J (JAX Stock No. 030898), B6.Cg-Ndor1 Tg(UBC-cre/ERT2)1Ejb/2J (JAX Stock No. 008085), B6.129-Psen1tm1Mpm/J (JAX Stock No. 004193) and C57BL/6NTac (JAX Stock No. 005703) mice were used. hAβ-KI (B6(SJL)-Apptm1.1Aduci/J) and WT (C57BL/6NTac) mice including both sexes (male and females) were analyzed at 2-, 6-, 10-, 14-, 18- and 22 months. Homozygous hAβ-KI (B6(SJL)-Apptm1.1Aduci/J) were crossed with homozygous PS1M146V-KI (B6.129-Psen1tm1Mpm/J), the resulting heterozygous hAβ-KI/PS1M146V mice were intercrossed to obtain homozygous knock-in mice hAβ-KI/ PS1M146V and aged to 18 months, both sexes were used. Homozygous hAβ-KI (B6(SJL)-Apptm1.1Aduci/J) were crossed with UBC-Cre-ERT2 (B6.Cg-Ndor1 Tg(UBC-cre/ERT2)1Ejb/2J) mice and the F1 animals (ApphAb-KI/+; UBC-Cre-ERT2 hemizygous) of both sexes were analyzed at 4-, 5- and 8 months. In addition, B6;129-Tg(APPSwe,tauP301L)1Lfa Psen1tm1Mpm/Mmjax mice (JAX Stock No. 34830) mice were used at 12-, 18- and 22-month of age as positive control in many experimental readouts.<br>Mice were housed in Super Mouse 750TM ventilated cages (Lab Products, Inc, Seaford, DE, USA). Cages contained 1/8" corn cob bedding (bed-o-cobs) and also contained 2 cotton nestlet squares for bedding. Lights were on a 12 h ON/OFF cycle, room temperature was set at 72oF with a variance of +/- 2 oF and ambient humidity conditions. A standard food diet (Envigo, Placentia, CA, USA; Ref 2020x) and water (purified by reverse osmosis) were provided ad libitum. |
| Wild animals | No wild animals were used |
| Field-collected samples | No field-collected samples were used |
| Ethics oversight | All animal procedures were conducted in accordance with the guidelines set forth by the National Institutes of Health and the University of California, Irvine (UCI) Institutional Animal Care and Use Committee (IACUC: AUP-17-38) who approved the study. |

Note that full information on the approval of the study protocol must also be provided in the manuscript.
